# Classification of *pmoA* amplicon pyrosequences using BLAST and the lowest common ancestor method in MEGAN

**DOI:** 10.3389/fmicb.2014.00034

**Published:** 2014-02-18

**Authors:** Marc G. Dumont, Claudia Lüke, Yongcui Deng, Peter Frenzel

**Affiliations:** ^1^Department of Biogeochemistry, Max Planck Institute for Terrestrial MicrobiologyMarburg, Germany; ^2^Department of Microbiology, Radboud UniversityNijmegen, Netherlands; ^3^Key Laboratory of Virtual Geographic Environment, Ministry of Education, Nanjing Normal UniversityNanjing, China

**Keywords:** *pmoA*, methanotroph, pyrosequencing, diversity, NGS data analysis

## Abstract

The classification of high-throughput sequencing data of protein-encoding genes is not as well established as for 16S rRNA. The objective of this work was to develop a simple and accurate method of classifying large datasets of *pmoA* sequences, a common marker for methanotrophic bacteria. A taxonomic system for *pmoA* was developed based on a phylogenetic analysis of available sequences. The taxonomy incorporates the known diversity of *pmoA* present in public databases, including both sequences from cultivated and uncultivated organisms. Representative sequences from closely related genes, such as those encoding the bacterial ammonia monooxygenase, were also included in the *pmoA* taxonomy. In total, 53 low-level taxa (genus-level) are included. Using previously published datasets of high-throughput *pmoA* amplicon sequence data, we tested two approaches for classifying *pmoA*: a naïve Bayesian classifier and BLAST. Classification of *pmoA* sequences based on BLAST analyses was performed using the lowest common ancestor (LCA) algorithm in MEGAN, a software program commonly used for the analysis of metagenomic data. Both the naïve Bayesian and BLAST methods were able to classify *pmoA* sequences and provided similar classifications; however, the naïve Bayesian classifier was prone to misclassifying contaminant sequences present in the datasets. Another advantage of the BLAST/LCA method was that it provided a user-interpretable output and enabled novelty detection at various levels, from highly divergent *pmoA* sequences to genus-level novelty.

## 1. Introduction

High-throughput sequencing (HTS) technologies have aided our ability to investigate the diversity of microorganisms in environmental samples either by shotgun metagenomic or amplicon sequencing approaches. Many bioinformatic tools necessary to process and interpret the large volume of data obtained by HTS have been developed. For example, there are several choices of pipelines available to analyze 16S rRNA amplicon sequencing data such as RDP (Cole et al., 2005), mothur (Schloss et al., 2009) and QIIME (Caporaso et al., 2010). Similar strategies targeting genes encoding enzymes responsible for important biogeochemical or bioremediation processes are becoming more common, but the methods for analyzing the data are not as well established as for 16S rRNA.

The analysis of HTS amplicon data can be performed using taxonomy-dependent or independent approaches. The taxonomy-independent approach includes methods to compare sequence alignments and analyze operational taxonomic units (OTUs) based on sequence dissimilarity (Schloss and Handelsman, 2004; Cai and Sun, 2011). This approach is valuable for estimating ecological parameters, such as richness and diversity; however, the information on its own does not indicate how the sequences are related to those of cultivated organisms or those from other studies. Taxonomy-based methods classify sequences according to their relatedness to those of pure cultures and uncultivated organisms. This approach is necessary to incorporate knowledge of the physiological characteristics of different taxa, to identify novel sequence types and to compare results between published studies. Common methods for classification include naïve Bayesian classifiers (Wang et al., 2007), k-nearest neighbor (Cole et al., 2005) and BLAST (Altschul et al., 1990).

In general, the analysis of OTUs and phylogenetic trees calculated from individual datasets of protein-encoding genes can be performed with the same tools designed for the analysis of 16S rRNA sequences. In contrast, classifiers must be tailor made for each gene by establishing a taxonomy with representative sequences and choosing an appropriate classification algorithm. The objective of this study was to establish a robust and easily applied approach to classifying HTS amplicon sequences of *pmoA*, a key gene of methane-oxidizing bacteria. The method should also allow for novelty detection and be easily performed by a microbial ecologist with only fundamental knowledge of bioinformatics. We test a naïve Bayesian classifier and BLAST combined with the lowest common ancestor approach of MEGAN using previously published *pmoA* pyrosequencing data (Lüke and Frenzel, 2011; Deng et al., 2013). Previous studies have also compared both approaches for the classification of SSU rRNA (Lanzén et al., 2012) and fungal LSU rRNA sequences (Porter and Golding, 2012).

## 2. *pmoA* taxonomy

An accurate taxonomic system for the gene sequences is a necessary prerequisite for classification. Since the classification of unknown sequences obtained by HTS can only be as accurate as the taxonomy, the analysis of database sequences and assignment of taxa is the critical step in the development of a classifier. In general, *pmoA* has been shown to be a good phylogenetic marker for methanotrophs (Degelmann et al., 2010), with some exceptions of divergent additional copies of the gene in some organisms (Dunfield et al., 2002; Stoecker et al., 2006; Baani and Liesack, 2008). Here we describe the taxonomy of *pmoA* genes (Table 1); earlier versions were described previously (Lüke and Frenzel, 2011; Deng et al., 2013).

**Table 1:** **Description of the *pmoA* database**.

**Classification level**	**MMO group designation**	**Cultivated representative**	**Database size (sequences)**	**Sequence representative**	**Typical habitats or origin**
0	Cu-containing membrane				
	Monooxygenase (CuMMO)		6628		
0.1	MOB_like				
0.1.1	TypeI				
0.1.1.1	TypeIa				
0.1.1.1.1	Mbacter	*Methylobacter tundripaludum* SV96	552	AJ414658	Arctic wetland
0.1.1.1.2	Mmicrobium_jap	*Methylomicrobium japanense*	28	AB253367	Marine mud
0.1.1.1.3	Mmicrobium_pel	*Methylomicrobium pelagicum* IR1	25	U31652	Upland soils
0.1.1.1.4	Mmonas	*Methylomonas methanica*	396	U31653	Lake sediments
0.1.1.1.5	Msarcina	*Methylosarcina quisquilarum*	517	AF177326	Landfill soil
0.1.1.1.6	Msoma	*Methylosoma difficile* LC2	4	DQ119047	Lake sediment
0.1.1.1.7	Mbacter_or_Mmonas		2	AY236078	Movile cave
0.1.1.1.8	Deep_sea_1		46	AM283467	Marine deep sea
0.1.1.1.9	Deep_sea_3		191	FJ858316	Marine deep sea
0.1.1.1.10	LP20		51	AB064377	Aquifer
0.1.1.1.11	Landfill_cluster_2		4	EU275117	Landfill soil
0.1.1.1.12	Lake_cluster_1		72	EF623667	Freshwater lakes
0.1.1.1.13	RPC_2		148	FN600101	Rice field soil
0.1.1.1.14	PS_80		8	AF211872	Marine
0.1.1.1.15	Aquifer_cluster		53	AM410175	Aquifer
0.1.1.2	TypeIb				
0.1.1.2.1	Mcaldum	*Methylocaldum tepidum*	98	U89304	Agricultural soil
0.1.1.2.2	Mcoccus	*Methylococcus capsulatus* Bath	244	L40804	Aquatic environments
0.1.1.2.3	Mthermus	*Methylothermus thermalis*	36	AJ829010	Hot spring
0.1.1.2.4	JRC_4	*Methylogaea oryzae*	27	EU359002	Rice field soil
0.1.1.2.5	Deep_sea_4		26	GU584280	Marine deep sea
0.1.1.2.6	Deep_sea_5		155	EU417471	Marine deep sea
0.1.1.2.7	FWs		100	AF211878	Freshwater lakes
0.1.1.2.8	JRC_3		29	AB222881	Rice field soil
0.1.1.2.9	Lake_cluster_2		74	AF211879	Freshwater lakes
0.1.1.2.10	LWs		83	DQ067069	Freshwater lakes
0.1.1.2.11	OSC		18	AJ317928	Organic soil
0.1.1.2.12	RPC_1		67	FN599957	Rice field soil
0.1.1.2.13	RPCs		166	FJ845814	Rice field soil
0.1.1.3	TypeIc				
0.1.1.3.1	Ncoccus	*Nitrosococcus oceani*	83	U96611	Marine
0.1.1.3.2	USCg		185	AJ579667	Upland soils
0.1.1.3.3	JR2		68	AY654695	Upland soils
0.1.1.3.4	JR3		65	AY654702	Upland soils
0.1.2	TypeII				
0.1.2.1	TypeIIa				
0.1.2.1.1	Msinus	*Methylosinus trichosporium* 33/1	70	AJ459007	Various
0.1.2.1.2	Mcystis	*Methylocystis* sp. strain SC2	1085	AJ431386	Various
0.1.2.1.3	Msinus_Mcystis	*Methylosinus trichosporium* str. KS21	79	AJ431388	Various
0.1.2.2	TypeIIb				
0.1.2.2.1	Mcapsa	*Methylocapsa acidiphila* B2	27	AJ278727	Sphagnum bog
0.1.2.2.2	MO3		23	AF283229	Landfill soil
0.1.2.2.3	pmoA2	*Methylocystis* sp. strain SC2	45	AJ431387	Various
0.1.2.2.4	USCa		888	AF148521	Upland soils
0.1.2	pXMO_like				
0.1.2.1	TUSC_like				
0.1.2.1.1	Verr_1	*Methylacidiphilum infernorum*	3	EU223859	Geothermal soil
0.1.2.1.2	Verr_2	*Methylacidiphilum infernorum*	3	EU223862	Geothermal soil
0.1.2.1.3	Verr_3	*Methylacidiphilum infernorum*	3	EU223855	Geothermal soil
0.1.2.1.4	TUSC		101	AJ868282	Various
0.1.2.1.5	NC10	Cand. Methylomirabilis oxyfera	33	JX262154	Freshwater sediment
0.1.2.2	RA21_like				
0.1.2.2.1	RA21		157	AF148522	Rice field soil
0.1.2.2.2	M84_P22		9	AJ299963	Rice field soil
0.1.2.2.3	gp23		1	AF264137	Upland soils
0.1.2.2.4	Alkane_1	Methylococcaceae ET-SHO	2	AB453961	Marine
0.1.2.2.5	Alkane_2	Methylococcaceae ET-HIRO	2	AB453962	Marine
0.1.2.2.6	MR1		7	AF200729	Upland soils
0.1.2.3	M84_P105_like				
0.1.2.3.1	M84_P105	*Methylomonas methanica*	34	EU722433	Various
0.1.2.4	Crenothrix_like				
0.1.2.4.1	Crenothrix	Crenothrix polyspora (enrichment)	69	DQ295904	Freshwater
0.1.2.4.2	Crenothrix_rel		160	AJ868245	Various
0.2	AOB_like	*Nitrosospira multiformis*	206	AF042171	Various

### 2.1. Overall taxonomic system

The *pmoA* gene encodes the β-subunit of the particulate methane monooxygenase (pMMO), which belongs to the class of copper-containing membrane-bound monooxygenase (CuMMO) enzymes. In addition to the pMMO, this group includes the bacterial ammonia monooxygenase (Holmes et al., 1995), the thaumarchaeal ammonia monooxygenase (Pester et al., 2011), alkane monooxygenases and various uncharacterized enzymes encoded by genes detected in environmental surveys (Coleman et al., 2012). For our classifier we compiled a database of *pmoA* and related gene sequences obtained primarily from public databases. We focused on building a taxonomic structure for *pmoA*, but also included sequences of related genes that are often co-amplified with common *pmoA* primers, such as the bacterial *amoA*. Related sequences that are not co-amplified, such as the thaumarchaeal *amoA*, were not included.

Currently, our curated database includes 6628 reference sequences corresponding to 53 low-level taxa (Table 1). The assignment to taxa was determined by the phylogenetic analysis of the *pmoA* and related gene fragments using both the nucleotide and inferred protein sequences. Sequences were imported into ARB (Ludwig et al., 2004) and alignments of either 408 nucleotide or 136 amino acid residues were used to generate neighbor-joining (NJ) and maximum-likelihood (ML) trees. For ML trees, sequences were exported and uploaded to the RAxML web-server (Stamatakis et al., 2005). Tree topologies were compared and taxa were assigned according to groups of sequences that consistently clustered together in the analyses (Lüke and Frenzel, 2011). At the highest level, the sequences were categorized as MOB_like or AOB_like, depending on apparent relatedness to sequences from methane-oxidizing and ammonia-oxidizing bacteria respectively. The classifier currently contains 53 low-level taxa within the MOB_like group (Table 1). Taxa comprising cultivated methanotrophs were referred to as the respective genera or species (e.g., Mbacter, for *Methylobacter*-like *pmoA*). Taxa lacking isolates were named according to representative clones or to the environment in which they were predominantly or initially found (e.g., Aquifer_cluster or upland soil cluster—USC) (Lüke and Frenzel, 2011).

### 2.2. Type I and II *pmoA* sequences

The MOB_like sequences were assigned to either Type I, Type II or pXMO_like. The Type I sequences were further divided into Type Ia, b, or c. Type Ia are *pmoA* sequences affiliated to the classic Type I methanotrophs (i.e., not Type X). Type Ib (also referred to elsewhere as Type X) are those of *Methylococcus* and closely related genera. Type Ic are all other Type I-related sequences with a more ambiguous affiliation. Type II sequences were divided into Type IIa and b. Type IIa was used for the primary *pmoA* sequences of the *Methylocystaceae*. Type IIb was used to group all other Type II-related (i.e., *Alphaproteobacteria*) sequences, including those from the *Beijerinckiaceae* (Theisen et al., 2005; Dunfield et al., 2010; Vorobev et al., 2011) and the alternate pMMO2 identified in some *Methylocystis* species (Dunfield et al., 2002; Baani and Liesack, 2008).

### 2.3. pXMO: divergent *pmoA* sequences

We use pXMO as the third category of *pmoA*-related sequences. The original description of pXMO was for the unusual pMMO-like enzyme identified in some Type I methanotrophs (Tavormina et al., 2011). Here we use pXMO_like to encompass all the divergent sequence types for which the substrate or biological function has not been clearly identified by biochemical or genetic tests. For example, we include the three verrucomicrobial *pmoA*-like sequences in this category until it is determined which, if not all, catalyze the oxidation of methane. The original *pxmA* genes identified in *Methylomonas* spp. (Tavormina et al., 2011) are classified in the M84_P105 low-level taxon. We have also included the *pmoA* sequences from the nitrite-dependent anaerobic methane oxidizers belonging to the NC10 phylum (Ettwig et al., 2009, 2010) into the pXMO_like category; it should be noted that these NC10 *pmoA* sequences are typically retrieved only after using specific primers and a special PCR program designed for their amplification (Luesken et al., 2011) and therefore are unlikely to be obtained in HTS *pmoA* surveys using the traditional *pmoA* primer sets.

### 2.4. Bacterial ammonia monooxygenase

Bacterial ammonia monooxygenase (*amoA*) genes were included since they are commonly co-amplified with *pmoA* genes in environmental PCR surveys. The *amoA* sequences of betaproteobacterial ammonia oxidizers were designated AOB_like, without making further lower-level distinctions. In contrast, the *amoA* sequences of *Nitrosococcus* were classified as “*Ncoccus*” within the MOB_like group since they are more closely related to *pmoA* than to other *amoA* genes.

## 3. Software and associated files

Mothur version 1.29.2 (Schloss et al., 2009) and MEGAN version 4.70.4 (Huson et al., 2011) were both downloaded from the author's webpages. Standalone BLAST 2.2.26+ (Camacho et al., 2009) was obtained from NCBI. These software programs can be installed on various platforms, but all analyses in this study were performed on a quad-core Apple MacBook Pro with a 2.2 GHz Intel Core i7 processor and 16 Gb of memory.

### 3.1. Naïve bayesian classifier

The training set for the naïve Bayesian classifier consists of two files: the database sequences in “fasta” format, and a taxonomy file indicating the taxonomy of each sequence. The taxonomy file was formatted to be compatible with mothur; both files are available in the supplement. The training set files were generated by exporting sequence information from ARB (Ludwig et al., 2004) and formatting the entries using standard spreadsheet and text-editing programs.

### 3.2. Blast and megan

The BLAST database was generated from the taxonomy using the makeblastdb program included with BLAST 2.2.26+ package. The input was a fasta file with the sequence name header including the sequence accession number and the taxon in square brackets; as for the naïve Bayesian classifier, these files were made using common spreadsheet and text-editing software. makeblastdb outputs three files (.nsq,.nin, and.nhr); all files are provided in the supplementary material.

A Newick format tree corresponding to the *pmoA* taxonomy was written for MEGAN; the tree file (pmoa.megan.2013.tre) and a corresponding map file (pmoa.megan.2013.map) are provided in the supplement. The *pmoA* taxonomy is loaded into MEGAN by the option “use alternative taxonomy” and selecting the pmoa.megan.2013.tre file.

## 4. *pmoA* amplification and sequencing

Two primer sets are typically used to amplify *pmoA* sequences from environmental samples (McDonald et al., 2008). The A189f/A682r primer pair offers broad specificity covering many CuMMO monooxygenases (Holmes et al., 1995). The A189f/mb661r combination was designed to be more specific for *pmoA* (Costello and Lidstrom, 1999) and does not generally amplify *amoA* or *pxmA*-like sequences. For NGS amplicon sequencing, adaptors and barcodes are incorporated into the primers, or ligated onto the PCR products, in a manner compatible with the sequencing platform (Binladen et al., 2007; Berry et al., 2011).

**Figure 1:**
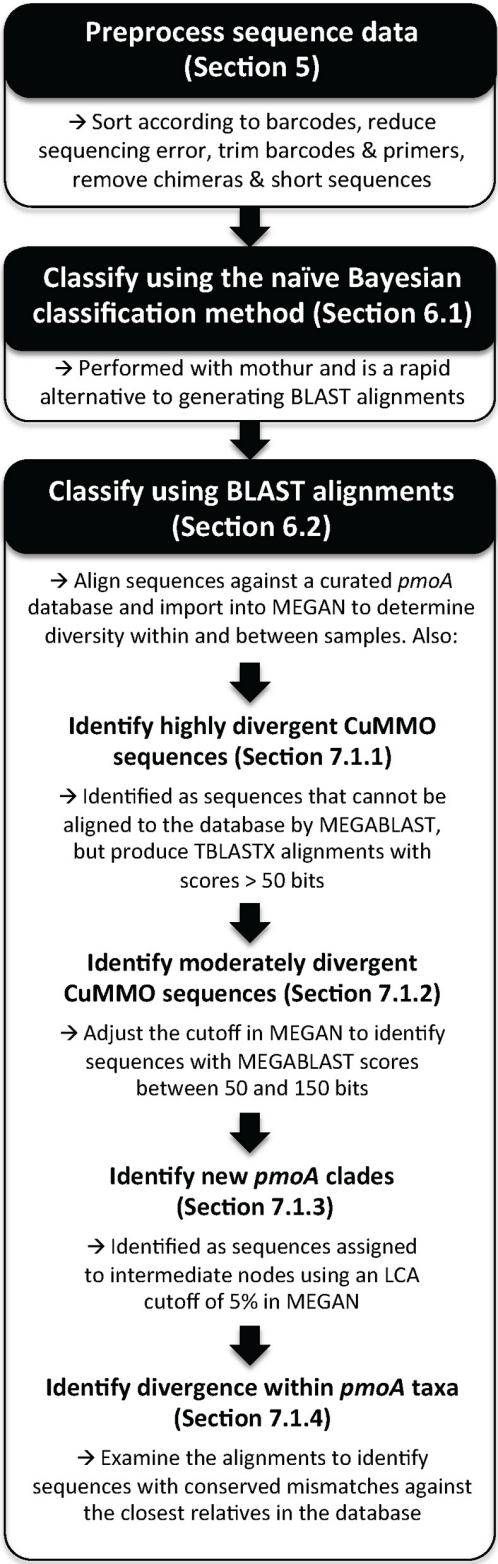
**Overview of the basic procedure for classifying *pmoA* sequences obtained by high-throughput sequencing**. The manuscript section where a procedure is described is indicated and detailed instructions are available in the supplementary materials.

Two previously described HTS *pmoA* amplicon datasets were used in this study to test the classification methods (Lüke and Frenzel, 2011; Deng et al., 2013). Both studies used the A189f/A682r and A189f/mb661r primer sets. The Lüke and Frenzel (2011) study focused on rice field soil samples from Italy and China and the sequences were analyzed in the original study by constructing phylogenetic trees and calculating OTUs from sequence alignments. The Deng et al. (2013) study focused on peatland samples from both submerged (hollow) and elevated (hummock) sites in the Qinghai-Tibetan plateau, and were analyzed in the original study by sequence similarity and an earlier implementation of the naïve Bayesian classifier. The basic steps for classifying *pmoA* sequence data are summarized in Figure 1 and a detailed protocol is included in the supplement.

## 5. Raw sequence processing

Raw sequence data must first undergo basic processing. In this study we use mothur, but other software can be used for this purpose, such as QIIME (Caporaso et al., 2010), RDP (Cole et al., 2009) and Funframe (Weisman et al., 2013); each of these have unique features that might be beneficial for a particular dataset or objective. The basic necessary steps are the sorting of sequences according to barcodes, trimming and quality filtering. For data analyzed here we use a minimum sequence length of 300 bp and remove sequences with ambiguities or stretches of more than eight homopolymers. Chimeric sequences were identified in mothur using the uchime method (Edgar et al., 2011). Chimeras were detected *de novo* by using the “self” option in mothur, meaning that the *pmoA* pyrosequencing dataset was used as a reference. As an example, in the HYa-1 dataset (Deng et al., 2013), 1058 chimeras were identified in 7658 sequences. These sequences were removed using the remove.seqs command in mothur (see supplementary methods).

NGS sequence technologies have relatively large error rates compared with Sanger sequencing and there are various approaches available to denoise pyrosequencing data (Reeder and Knight, 2010; Quince et al., 2011; Rosen et al., 2012). If not removed, these errors generate false OTUs (Schloss et al., 2011). Another problem is that they often cause frameshift errors in protein-coding genes, making it difficult to infer amino acid sequences. There are methods available to specifically correct frameshift errors in functional gene pyrosequencing datasets, such as the FrameBot tool (http://fungene.cme.msu.edu/FunGenePipeline/) and HMM-FRAME (Weisman et al., 2013). Correcting the frameshift errors in pyrosequencing datasets is particularly important for calculating OTUs or phylogenetic distances based on amino acid sequences. In general we did not find denoising necessary for classifying our *pmoA* sequences obtained by pyrosequencing and therefore do not discuss the methods here; however, a study on fungal LSU rRNA amplicon sequencing reported that the BLAST/LCA method was less sensitive to sequence error than the naïve Bayesian classification approach (Porter and Golding, 2012).

## 6. Classification of *pmoA* sequences

### 6.1. Naïve bayesian classifier

A naïve Bayesian classifier is the basis of the RDP classifier for 16S rRNA sequence data (Wang et al., 2007). In this study we have adopted the 16S rRNA classifier implemented in mothur, but replaced the 16S rRNA taxonomy with that of *pmoA*. The process in mothur is invoked with classify.seqs command with the option of the “wang” method. Other variables include the kmer (word) size and the cutoff value for bootstrap confidence estimates. In general we found that the default kmer size of 8 performed well and used a cutoff value of 80%.

### 6.2. BLAST/LCA

Different versions of BLAST were tested. Nucleotide BLAST types include MEGABLAST, BLASTN and discontiguous (DC)-MEGABLAST, in decreasing order of sensitivity for obtaining matches and alignments against distantly related sequences (McGinnis and Madden, 2004). In most cases the results of the different methods were nearly identical, except in some cases that DC-MEGABLAST would produce hits (alignments) with distantly related novel *pmoA* sequences that were not aligned by the other two methods. We found that the additional computation time required for DC-MEGABLAST was not compensated by the added sensitivity since this could be easily recovered by a reanalysis of the sequences that did not produce hits with MEGABLAST, as described in section 7.1.1. Protein BLAST approaches were also tested and classification results were similar to nucleotide BLAST, but with greater sensitivity for matching distantly related novel sequence types to the database. The protein BLAST searches were more vulnerable to the effect of frameshift errors in the query sequences because they cause breaks in the alignment that strongly affect the bit score of a match. In contrast, the insertion of gap during nucleotide BLAST adds a small penalty to the bit score and does not terminate the alignment. Therefore, in general we use MEGABLAST, which is the default algorithm for the blastn program.

BLAST has been shown to be relatively poor at identifying the most similar sequence in a dataset (Koski and Golding, 2001; Cole et al., 2005). We did not observe, nor do we foresee, this to be a problem for the classification since it is only necessary to identify the most similar taxon, which are all highly distinct (>5% nucleic acid identity) from one another.

#### 6.2.1. BLAST interpretation by lowest common ancestor in MEGAN

MEGAN was developed for the classification of metagenomic sequence data by reading the output of BLAST queries (Huson et al., 2011; Mitra et al., 2010). It has been adapted for other purposes, for example it can read the log file of a sina alignment of 16S rRNA (Pruesse et al., 2012) and thereby used to analyze 16S rRNA sequence data (Mitra et al., 2011). Here we show that it could be adapted for the analysis of the *pmoA* BLAST queries against our taxonomy database, as has been demonstrated previously for SSU rRNA (Lanzén et al., 2012) and fungal LSU rRNA (Porter and Golding, 2012). A simple modification is possible in MEGAN to change the default NCBI taxonomy to a custom taxonomy, in this case *pmoA*. MEGAN parses the BLAST output file and collects only the top hits from each taxon and the associated alignment. In addition to summarizing the results, this has the added benefit of reducing the file size compared with the original BLAST output.

MEGAN uses a lowest common ancestor (LCA) algorithm (Huson et al., 2007) based on BLAST bit scores to classify the sequences. A sequence is classified at a particular level only when the bit score to the taxon is higher by a given margin than to those of any another taxon. The margin of difference can be adjusted in the LCA parameters of MEGAN with the option of “top percent.” The greater the margin, the greater is the minimum distance between the assigned taxon and any other taxon. The BLAST/LCA method provides several valuable benefits for the classification in comparison to simply classifying based on top hit, for example in novelty detection as discussed below.

### 6.3. Comparison of the Naïve Bayesian and BLAST/LCA classifications

**Figure 2:**
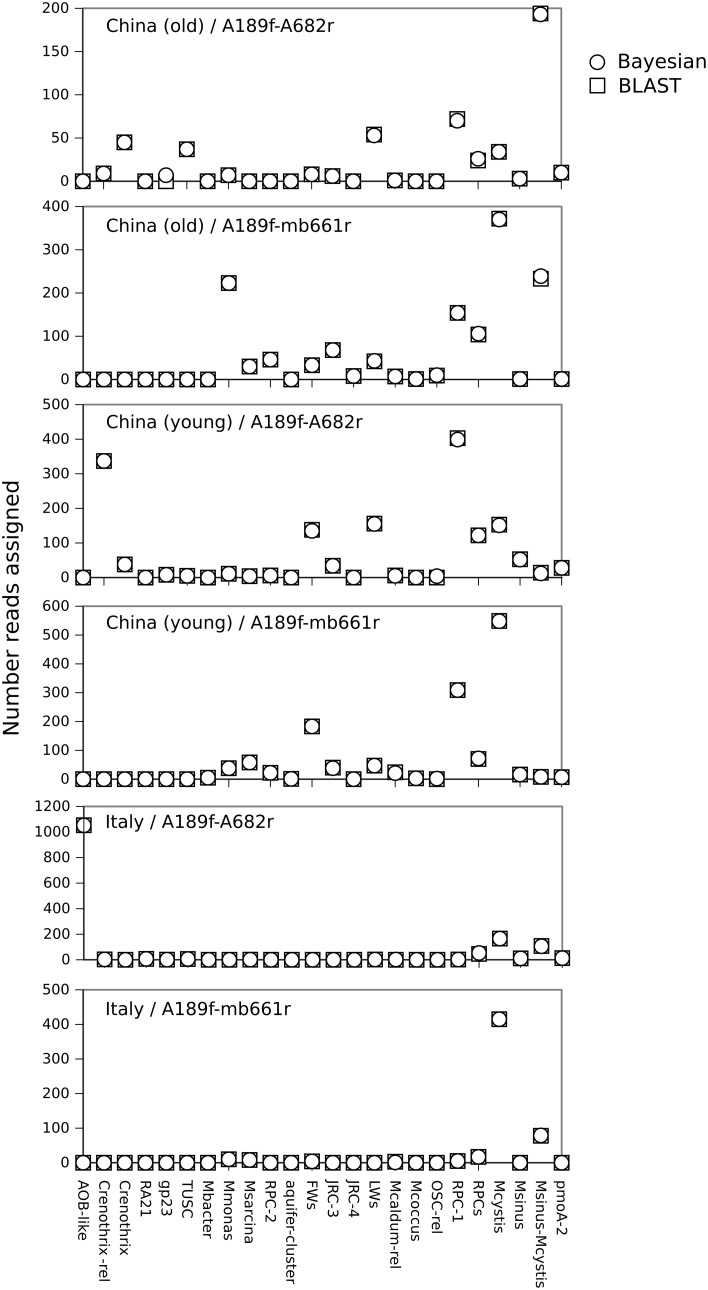
**Comparison of classifications of paddy soil *pmoA* sequence data (Lüke and Frenzel, 2011) using the naïve Bayesian and BLAST/LCA methods**. The datasets were generated from three different soils (young Chinese, old Chinese, and Italian) and with two different PCR primer combinations (A189f/A682r, A189f/mb661r) as indicated. The number of sequences assigned to each taxon is plotted. Only *pmoA* taxa detected in at least one dataset are shown.

We found good agreement in the classification of the rice paddy soil datasets using the naïve Bayesian and BLAST/LCA approaches (Figure 2). Subsamples from each classification were analyzed in ARB by NJ and in general confirmed the assignments (results not shown). Some minor differences between the methods were also observed. For example, seven sequences in the China (old) A189f-A682r dataset were classified as gp23 whereas these seven sequences did not produce significant hits using BLAST/LCA. A close inspection indicated that the seven sequences were either highly divergent *pmoA* or non-specific PCR products related to an alpha-glucan branching protein or a peptidase. An analysis of the Riganqiao samples also showed a tendency for the naïve Bayesian classifier to assign contaminant sequences to gp23 (not shown). This is likely a result of gp23 only being represented by a single sequence in the database and being relatively divergent from other *pmoA* taxa (Lüke and Frenzel, 2011). This erroneous classification of non-target sequences as gp23 using the naïve Bayesian classifier could be reduced by increasing the kmer size to 10, but at a cost of decreased sensitivity in classifying bona fide *pmoA* sequences. In spite of this, both the naïve Bayesian and BLAST/LCA methods identified genuine gp23 sequences present in the China (young) A189f-A682r dataset (Figure 2). Previous studies also reported higher accuracies of classifications obtained by BLAST/LCA for 16S rRNA sequences (Lanzén et al., 2012), fungal LSU rRNA sequences (Porter and Golding, 2012) and rRNA internal transcribed spacer sequences (Porter and Golding, 2011). However, one clear advantage of the naïve Bayesian classifier was speed; on our system it could classify thousands of *pmoA* sequences per second compared to approximately 200 sequences per min for the MEGABLAST query.

## 7. Novelty detection

Novel sequences can be identified by the naïve Bayesian classifier if they cannot be classified. For example, a novel taxon within the Type Ib's should be returned as “unclassified Type Ib.” It is possible to adjust this further by specifying a percent cutoff value for a bootstrapped analysis. In general, we found this method to be unreliable as even contaminant sequences were classified as gp23 with >80% bootstrap values, as discussed above.

### 7.1. Novelty detection using BLAST/LCA in MEGAN

The BLAST/LCA procedure offers approaches for novelty detection at various levels, which are described individually in the following sections.

#### 7.1.1. Identification of highly divergent CuMMO sequences

Deeply-branching novel sequences did not produce hits to the database, which is the first indication that it is potentially a novel sequence clade. This occurs in BLAST queries when the sequence does not contain a match of the minimum word size, which is 28 nucleotides in MEGABLAST. For example, this was the case in the Riganqiao dataset with the novel clade that was termed HY-3 (Deng et al., 2013). In contrast to MEGABLAST, these sequences could be identified as *pmoA* by a translated BLAST query (TBLASTX). The difference is that protein sequences are more conserved than nucleotide sequences and BLASTX uses a word size of 3 amino acids compared with a minimum word size of 7 nucleotides for BLASTN. Therefore, the first step in novelty detection is to select the sequences that do not produce MEGABLAST hits and to query them against the database using TBLASTX; in our experience, highly divergent novel clusters will produce significant hits (>50 bits) with TBLASTX, whereas unrelated contaminant sequences will not.

#### 7.1.2. Identification of moderately divergent CuMMO sequences

Moderately divergent novel sequence clades can be identified by a relatively low MEGABLAST bit score. The bit score cutoffs can be adjusted in MEGAN and here we used a threshold of 150 bits to identify new clades. An example of a novel sequence that could be identified in this manner was the I141NRXW sequence from the paddy soil (Lüke and Frenzel, 2011), which had a top score of 89.8 bits. In comparison, the sequence with the next lowest bit score in that dataset had a value of 374, and maximum bit scores approached 900.

#### 7.1.3. Lowest common ancestor classification of sequences

**Figure 3:**
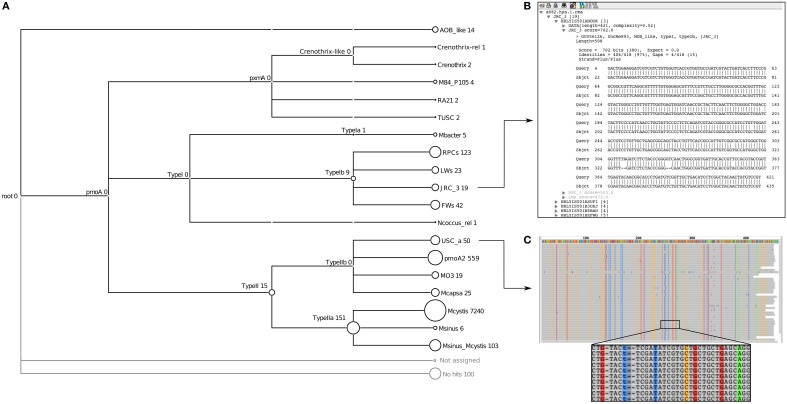
**Examples of MEGAN visualizations of assignments for the *pmoA* data of the HYa-1 sample obtained from the Riganqiao *pmoA* pyrosequencing study (Deng et al., 2013)**. The tree shows the summary of taxa identified and their abundances **(A);** the circles at the nodes are proportional to the number of reads assigned to that taxon. Individual assignments can be inspected by right-clicking on a node and selecting “inspect,” as shown for the JRC-3 clade **(B)**. Summary alignments can also be visualized, as shown for USCα **(C);** in this case it quickly shows that the best alignment to USCα have approximately 40 conserved mismatches, suggesting it is a novel *pmoA* cluster most closely related to USCα.

**Figure 4:**
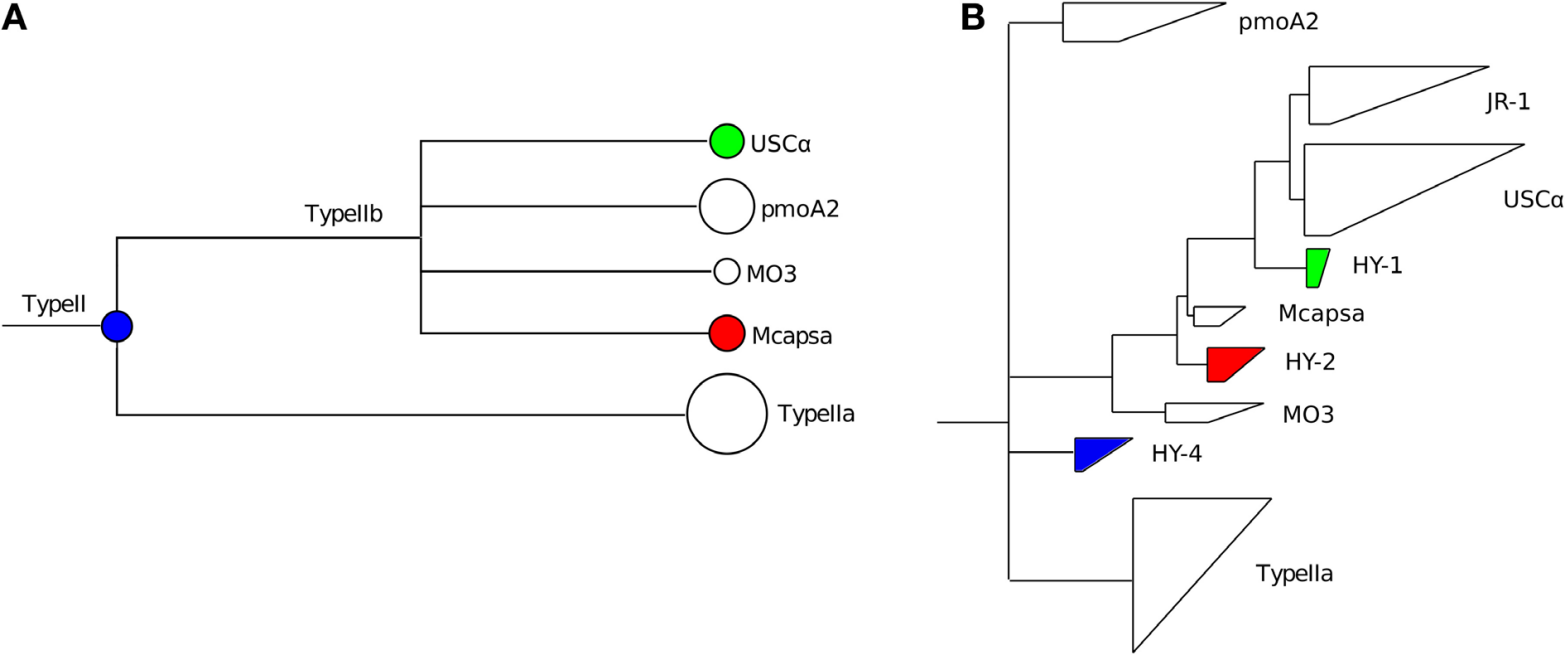
**Analysis of selected Type II *pmoA* sequences from the HYa-1 sample (Deng et al., 2013)**. The sequences chosen for analysis are color-coded in the partial MEGAN tree **(A)**. The USCα and *Methylocapsa*-assigned sequences were selected since the alignments showed conserved mismatches to the reference database (as shown for USCα sequences in Figure 3C). The sequences were imported into an ARB *pmoA* database, quality filtered by removing sequences with frameshifts, translated to amino acid sequences and added to the *PmoA* tree by parsimony and then reanalyzed by neighbor-joining. The positions of the sequences analyzed in ARB are shown **(B)**. The new clades were named HY-1, HY-2 (Deng et al., 2013) and HY-4 (this study).

The next level of novelty can be identified using the lowest common ancestor (LCA) algorithm in MEGAN. For the HYa-1 dataset, assignments to higher nodes could be seen at the Type II (15 sequences), Type Ib (9 sequences) and Type IIa (151 sequences) taxonomic levels using a margin of 5% for the LCA calculation (Figure 3A). In contrast, classifications to the other lowest-level taxa were stable even using an LCA margin greater than 25%. The inability to classify sequences to the lowest levels indicates that the sequence may represent a new taxon branching from the LCA node. For example, the sequences assigned to the Type II node had similar bit scores to both *Methylocystis* and *pmoA2* clades and a NJ analysis of the sequences also suggested that it was new lineage (termed HY-4) at the root of the Type II's (Figure 4). The assignments at the node of the Type IIa branch were the result of an inability to distinguish between the *Methylocystis* and *Methylosinus*-*Methylocystis* taxa, which might indicate intermediate sequence types or the existence of a bush-like continuum in this region of the tree (Lüke and Frenzel, 2011).

In addition to biological diversity, there could also be technical errors that impede classification, such as sequencing noise or chimerism. Furthermore, falsely assigned sequences in the taxonomy file would also result in sequences failing to be classified at the lowest level. Both of these situations can generally be detected by visually analyzing the BLAST alignments, as described in the following section.

#### 7.1.4. Lowest-level diversity: examination of hits and alignments

The final level of novelty can be detected by examining the hits and alignments against the database. BLAST alignments of individual reads (Figure 3B) or summary alignments for groups of sequences (Figure 3C) can be examined in MEGAN. Examining individual reads gives an impression of how closely related a sequence is to members of the assigned taxon compared to the next-nearest taxon. In the example shown (Figure 3B), the top hit had 97% identity to a JRC_3 (702 bits) and the next best hit of only 89% identity (272 bits) to RPC_1. In this example, it is evident that the sequence is genuinely JRC_3. In contrast, the hits to *Methylocapsa* had maximum identities of only 95% (not shown), suggesting that these might represent a closely related novel cluster. An analysis by NJ of these sequences indicated that indeed they formed a new branch close to *Methylocapsa* (termed HY-2) (Figure 4).

The second option is to invoke the alignment of the hits to a taxon. Here it is possible to see evidence of novelty within a group of sequences classified to a particular taxon. For example, in some cases numerous conserved mismatches against the top database reference can suggest that the sequences belong to a divergent clade mostly related to the assigned taxon. For example, about 40 conserved mismatches were present within the assignment to USCα in the HYa-1 dataset (Figure 3C). A closer analysis by NJ of these sequences indicated they were a novel lineage most closely related to USCα (termed HY-1) (Figure 4).

## 8. Data comparisons and downstream analysis

**Figure 5:**
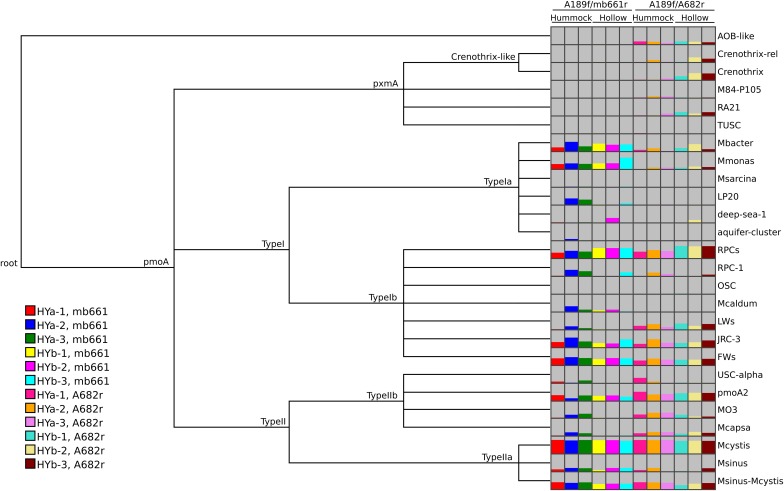
**MEGAN comparison view of *pmoA* classifications from the Riganqiao *pmoA* pyrosequencing datasets (Deng et al., 2013)**. The *pmoA* datasets were obtained from triplicate samples from hummock (HYa) and hollow (HYb) sites. PCRs were performed with two primer combinations (A189f/A682r or A189f/mb661r), as indicated. The option to subsample datasets (3309 sequences) was chosen for the comparison. Assignments to internal nodes are not shown. MEGAN only shows taxa detected in at least one sample. The height of the bars was scaled to the number of reads assigned in each dataset and color-coded as indicated in the legend. The labeling at the top of the columns was added.

MEGAN has several built-in functions that offer possibilities to visualize and analyze the results. Comparisons between samples can be easily made using various visualization options. Trends in the data can be observed and demonstrated, such as the relative coverage of the two primer sets and the elevated abundance of RPCs in hollow soils (Figure 5). MEGAN can also calculate matrices of pairwise distances using six ecological measures: Euclidian, Goodall, Chi-Square, Kulczynski, Bray-Curtis, and Hellinger (Mitra et al., 2010). Data can also be easily exported into statistical software programs. Of course, the classification of sequence data to taxa is only one step in the analysis of HTS amplicon data of protein-coding genes and should be complemented by classification-independent analyses.

## 9. Conclusions

Although the naïve Bayesian and BLAST/LCA methods provided similar classifications of the high-throughput *pmoA* sequence data examined in this study, the BLAST/LCA approach had several advantages, such as being less sensitive to false classification of contaminant sequences and offering several options for novelty detection at various levels of sequence divergence. The BLAST/LCA method has another advantage that a researcher can visually interpret the calculations, in the form of alignments, therefore enabling the results to be verified and judged.

### Conflict of interest statement

The authors declare that the research was conducted in the absence of any commercial or financial relationships that could be construed as a potential conflict of interest.

## References

[B1] Altschul S. F. Gish W. Miller W. Myers E. W. Lipman D. J. (1990). Basic local alignment search tool. J. Mol. Biol. 215, 403–410 10.1006/jmbi.1990.9999 2231712

[B2] Baani M. Liesack W. (2008). Two isozymes of particulate methane monooxygenase with different methane oxidation kinetics are found in *Methylocystis* sp strain SC2. Proc. Natl. Acad. Sci. U.S.A. 105, 10203–10208 10.1073/pnas.0702643105 18632585 PMC2481331

[B3] Berry D. Ben Mahfoudh K. Wagner M. Loy A. (2011). Barcoded primers used in multiplex amplicon pyrosequencing bias amplification. Appl. Environ. Microbiol. 77, 7846–7849 10.1128/AEM.05220-11 21890669 PMC3209180

[B4] Binladen J. Gilbert M. T. P. Bollback J. P. Panitz F. Bendixen C. Nielsen R. (2007). The use of coded PCR primers enables high-throughput sequencing of multiple homolog amplification products by 454 parallel sequencing. PLoS ONE 2:e197 10.1371/journal.pone.0000197 17299583 PMC1797623

[B5] Cai Y. Sun Y. (2011). ESPRIT-Tree: hierarchical clustering analysis of millions of 16S rRNA pyrosequences in quasilinear computational time. Nucleic Acids Res. 2011, 1–10 10.1093/nar/gkr349 21596775 PMC3152367

[B6] Camacho C. Coulouris G. Avagyan V. Ma N. Papadopoulos J. Bealer K. (2009). BLAST+: architecture and applications. Bioinformatics 10, 421 10.1186/1471-2105-10-421 20003500 PMC2803857

[B7] Caporaso J. G. Kuczynski J. Stombaugh J. Bittinger K. Bushman F. D. Costello E. K. (2010). QIIME allows analysis of high-throughput community sequencing data. Nat. Methods 7, 335–336 10.1038/nmeth.f.303 20383131 PMC3156573

[B8] Cole J. R. Chai B. Farris R. J. Wang Q. Kulam S. A. McGarrell D. M. (2005). The Ribosomal Database Project (RDP-II): sequences and tools for high-throughput rRNA analysis. Nucleic Acids Res. 33, D294–D296 10.1093/nar/gki038 15608200 PMC539992

[B9] Cole J. R. Wang Q. Cardenas E. Fish J. Chai B. Farris R. J. (2009). The Ribosomal Database Project: improved alignments and new tools for rRNA analysis. Nucleic Acids Res. 37, D141–D145 10.1093/nar/gkn879 19004872 PMC2686447

[B10] Coleman N. V. Le N. B. Ly M. A. Ogawa H. E. McCarl V. Wilson N. L. (2012). Hydrocarbon monooxygenase in *Mycobacterium*: recombinant expression of a member of the ammonia monooxygenase superfamily. ISME J. 6, 171–182 10.1038/ismej.2011.98 21796219 PMC3246247

[B11] Costello A. M. Lidstrom M. E. (1999). Molecular characterization of functional and phylogenetic genes from natural populations of methanotrophs in lake sediments. Appl. Environ. Microbiol. 65, 5066–5074 10543824 10.1128/aem.65.11.5066-5074.1999 PMC91682

[B12] Degelmann D. M. Borken W. Drake H. L. Kolb S. (2010). Different atmospheric methane-oxidizing communities in European beech and Norway spruce soils. Appl. Environ. Microbiol. 76, 3228–3235 10.1128/AEM.02730-09 20348309 PMC2869149

[B13] Deng Y. Cui X. Lüke C. Dumont M. G. (2013). Aerobic methanotroph diversity in Riganqiao peatlands on the Qinghai-Tibetan Plateau. Environ. Microbiol. Rep. 5, 566–574 10.1111/1758-2229.12046 23864571

[B14] Dunfield P. F. Belova S. E. Vorob'ev A. V. Cornish S. L. Dedysh S. N. (2010). *Methylocapsa aurea* sp. nov., a facultative methanotroph possessing a particulate methane monooxygenase, and emended description of the genus Methylocapsa. Int. J. Syst. Evol. Microbiol. 60, 2659–2664 10.1099/Ijs.0.020149-0 20061505

[B15] Dunfield P. F. Yimga M. T. Dedysh S. N. Berger U. Liesack W. Heyer J. (2002). Isolation of a *Methylocystis* strain containing a novel *pmoA*-like gene. FEMS Microbiol. Ecol. 41, 17–26 10.1111/j.1574-6941.2002.tb00962.x 19709235

[B16] Edgar R. C. Haas B. J. Clemente J. C. Quince C. Knight R. (2011). UCHIME improves sensitivity and speed of chimera detection. Bioinformatics 27, 2194–2200 10.1093/bioinformatics/btr381 21700674 PMC3150044

[B17] Ettwig K. F. Butler M. K. Le Paslier D. Pelletier E. Mangenot S. Kuypers M. M. M. (2010). Nitrite-driven anaerobic methane oxidation by oxygenic bacteria. Nature 464, 543–548 10.1038/nature08883 20336137

[B18] Ettwig K. F. van Alen T. van de Pas-Schoonen K. T. Jetten M. S. M. Strous M. (2009). Enrichment and molecular detection of denitrifying methanotrophic bacteria of the NC10 phylum. Appl. Environ. Microbiol. 75, 3656–3662 10.1128/aem.00067-09 19329658 PMC2687271

[B19] Holmes A. J. Costello A. Lidstrom M. E. Murrell J. C. (1995). Evidence that particulate methane monooxygenase and ammonia monooxygenase may be evolutionarily related. FEMS Microbiol. Lett. 132, 203–208 10.1111/j.1574-6968.1995.tb07834.x 7590173

[B20] Huson D. H. Auch A. F. Qi J. Schuster S. C. (2007). MEGAN analysis of metagenomic data. Genome Res. 17, 377–386 10.1101/gr.5969107 17255551 PMC1800929

[B21] Huson D. H. Mitra S. Ruscheweyh H. J. Weber N. Schuster S. C. (2011). Integrative analysis of environmental sequences using MEGAN4. Genome Res. 21, 1552–1560 10.1101/gr.120618.111 21690186 PMC3166839

[B22] Koski L. B. Golding G. B. (2001). The closest BLAST hit is often not the nearest neighbor. J. Mol. Evol. 52, 540–542 10.1007/s002390010184 11443357

[B23] Lanzén A. Jørgensen S. L. Huson D. H. Gorfer M. Grindhaug S. H. Jonassen I. (2012). CREST – Classification resources for environmental sequence tags. PLoS ONE 77:e49334 10.1371/journal.pone.0049334 23145153 PMC3493522

[B24] Ludwig W. Strunk O. Westram R. Richter L. Meier H. Yadhukumar (2004). ARB: a software environment for sequence data. Nucleic Acids Res. 32, 1363–1371 10.1093/nar/gkh293 14985472 PMC390282

[B25] Luesken F. A. Zhu B. van Alen T. A. Butler M. K. Rodriguez Diaz M. Song B. (2011). *pmoA* primers for detection of anaerobic methanotrophs. Appl. Environ. Microb. 77, 3877–3880 10.1128/AEM.02960-10 21460105 PMC3127593

[B26] Lüke C. Frenzel P. (2011). Potential of *pmoA* amplicon pyrosequencing for methanotroph diversity studies. Appl. Environ. Microb. 77, 6305–6309 10.1128/Aem.05355-11 21764977 PMC3165393

[B27] McDonald I. R. Bodrossy L. Chen Y. Murrell J. C. (2008). Molecular ecology techniques for the study of aerobic methanotrophs. Appl. Environ. Microb. 74, 1305–1315 10.1128/Aem.02233-07 18165358 PMC2258629

[B28] McGinnis S. Madden T. L. (2004). BLAST: at the core of a powerful and diverse set of sequence analysis tools. Nucleic Acids Res. 32, W20–W25 10.1093/nar/gkh435 15215342 PMC441573

[B29] Mitra S. Gilbert J. A. Field D. Huson D. H. (2010). Comparison of multiple metagenomes using phylogenetic networks based on ecological indices. ISME J. 4, 1236–1242 10.1038/ismej.2010.51 20428222

[B30] Mitra S. Stark M. Huson D. H. (2011). Analysis of 16S rRNA environmental sequences using MEGAN. BMC Genomics 12 10.1186/1471-2164-12-s3-s17 22369513 PMC3333176

[B31] Pester M. Schleper C. Wagner M. (2011). The Thaumarchaeota: an emerging view of their phylogeny and ecophysiology. Curr. Opin. Microbiol. 14, 300–306 10.1016/j.mib.2011.04.007 21546306 PMC3126993

[B32] Porter T. M. Golding G. B. (2011). Are similarity- or phylogeny-based methods more appropriate for classifying internal transcribed spacer (ITS) metagenomic amplicons? New Phytol. 192, 775–782 10.1111/j.1469-8137.2011.03838.x 21806618

[B33] Porter T. M. Golding G. B. (2012). Factors that affect large subunit ribosomal DNA amplicon sequencing studies of fungal communities: classification method, primer choice, and error. PLoS ONE 7:e35749 10.1371/journal.pone.0035749 22558215 PMC3338786

[B34] Pruesse E. Peplies J. Glöckner F. O. (2012). SINA: accurate high-throughput multiple sequence alignment of ribosomal RNA genes. Bioinformatics 28, 1823–1829 10.1093/bioinformatics/bts252 22556368 PMC3389763

[B35] Quince C. Lanzen A. Davenport R. J. Turnbaugh P. J. (2011). Removing noise from pyrosequenced amplicons. BMC Bioinform. 12:38 10.1186/1471-2105-12-38 21276213 PMC3045300

[B36] Reeder J. Knight R. (2010). Rapidly denoising pyrosequencing amplicon reads by exploiting rank-abundance distributions. Nat. Methods 7:668–669 10.1038/nmeth0910-668b 20805793 PMC2945879

[B37] Rosen M. J. Callahan B. J. Fisher D. S. Holmes S. P. (2012). Denoising PCR-amplified metagenome data. BMC Bioinform. 13:283 10.1186/1471-2105-13-283 23113967 PMC3563472

[B38] Schloss P. D. Handelsman J. (2004). Status of the microbial census. Microbiol. Mol. Biol. Rev. 68, 686–691 10.1128/mmbr.68.4.686-691.2004 15590780 PMC539005

[B39] Schloss P. D. Westcott S. L. Ryabin T. Hall J. R. Hartmann M. Hollister E. B. (2009). Introducing mothur: open-source, platform-independent, community-supported software for describing and comparing microbial communities. Appl. Environ. Microbiol. 75, 7537–7541 10.1128/aem.01541-09 19801464 PMC2786419

[B40] Schloss P. D. Gevers D. Westcott S. L. (2011). Reducing the effects of PCR amplification and sequencing artifacts on 16S rRNA-based studies. PLoS ONE 6:e27310 10.1371/journal.pone.0027310 22194782 PMC3237409

[B41] Stamatakis A. Ludwig T. Meier H. (2005). RAxML-III: a fast program for maximum likelihood-based inference of large phylogenetic trees. Bioinformatics 21, 456–463 10.1093/bioinformatics/bti191 15608047

[B42] Stoecker K. Bendinger B. Schoning B. Nielsen P. H. Nielsen J. L. Baranyi C. (2006). *Cohn's Crenothrix* is a filamentous methane oxidizer with an unusual methane monooxygenase. Proc. Natl. Acad. Sci. U.S.A. 103, 2363–2367 10.1073/pnas.0506361103 16452171 PMC1413686

[B43] Tavormina P. L. Orphan V. J. Kalyuzhnaya M. G. Jetten M. S. M. Klotz M. G. (2011). A novel family of functional operons encoding methane/ammonia monooxygenase-related proteins in gammaproteobacterial methanotrophs. Environ. Microbiol. Rep. 3, 91–100 10.1111/j.1758-2229.2010.00192.x 23761236

[B44] Theisen A. R. Ali M. H. Radajewski S. Dumont M. G. Dunfield P. F. McDonald I. R. (2005). Regulation of methane oxidation in the facultative methanotroph *Methylocella silvestris* BL2. Mol. Microbiol. 58, 682–692 10.1111/j.1365-2958.2005.04861.x 16238619

[B45] Vorobev A. V. Baani M. Doronina N. V. Brady A. L. Liesack W. Dunfield P. F. (2011). *Methyloferula stellata* gen. nov., sp nov., an acidophilic, obligately methanotrophic bacterium that possesses only a soluble methane monooxygenase. Int. J. Syst. Evol. Microbiol. 61, 2456–2463 10.1099/ijs.0.028118-0 21097638

[B46] Wang Q. Garrity G. M. Tiedje J. M. Cole J. R. (2007). Naïve Bayesian classifier for rapid assignment of rRNA sequences into the new bacterial taxonomy. Appl. Environ. Microbiol. 73, 5261–5267 10.1128/aem.00062-07 17586664 PMC1950982

[B47] Weisman D. Yasuda M. Bowen J. L. (2013). FunFrame: functional gene ecological analysis pipeline. Bioinformatics 29, 1212–1214 10.1093/bioinformatics/btt123 23511542

