# Supplementary material for: Classification of pmoA amplicon pyrosequences using BLAST and the lowest common ancestor method in MEGAN
Source: Front Microbiol. 2014 Feb 18;5:34. doi: 10.3389/fmicb.2014.00034 (PMC3927136; doi:10.3389/fmicb.2014.00034)
Supplement: Supplementary file 1 [file Presentation1.ZIP › Supplementary Materials 10.3389 fmicb.2014.00034/method overview.pdf]

Basic steps for analyzing a *pmoA* pyrosequencing dataset

1. Download and install software:

- mothur (http://www.mothur.org/wiki/Download_mothur)
- standalone blast (ftp://ftp.ncbi.nlm.nih.gov/blast/executables/blast+/LATEST/)
- MEGAN (http://ab.inf.uni-tuebingen.de/software/megan/)

2. Obtain *pmoA* classifier files (this study):

- Naïve Bayesian classifier (pmoa.mothur.2013.fasta, pmoa.mothur.2013.tax)
- BLAST database (pmoa.nhr, pmoa.nin, pmoa.nsq); I also included the original file (pmoA.blast.db.2013.txt) so that you can modify the database and rebuild it using the makeblastdb program in the blast suite.
- MEGAN files (pmoa.megan.2013.tre, pmoa.megan.2013.map)

3. Preprocess *pmoA* 454 sequences (sff file format) using mothur (Schloss et al., 2009):

a) Extract files from *your.pmoa*.sff using sffinfo, e.g. as follows:
   sffinfo(sff=*your.pmoa*.sff, flow=T)
b) Sort sequences according to your barcodes using trim.flows, e.g. as follows:
   trim.flows(flow=*your.pmoa*.flow, oligos=*your*.oligos, pdiffs=2, bdiffs=1, order=B, processors=8)
   -- Note 1: check the flow order that was used for the run. The default is A, but now most sequencing with 454 uses the acyclic order (indicated in mothur as order=B).
   -- Note 2: make your own oligos file corresponding to the primers, barcodes, linkers used for your project. You can get a description of the format and find examples in the mothur wiki.
c) Reduce sequencing errors using shhh.flows, e.g. as follows:
   shhh.flows(file=*your.pmoa*.flow.files, processors=8)
   -- Note: you need to download LookUp.Titanium.pat file from the mothur website
d) Remove barcodes, primers and set minimum sequence length using trim.seqs, e.g. as follows:
   trim.seqs(fasta=*your.pmoa*.shhh.fasta, name=*your.pmoa*.shhh.names, oligos=your.oligos, pdiffs=2, bdiffs=1, minlength=200, processors=8)
e) Identify potential chimeras, as follows:
   chimera.uchime(fasta=*your.pmoa*.shhh.trim.fasta, name=*your.pmoa*.shhh.trim.names, group=*your.pmoa*.shhh.groups, reference=self, processors=8)
f) Remove chimeras from your dataset:
   remove.seqs(accnos=*your.pmoa*.shhh.trim.uchime.accnos, fasta=*your.pmoa*.shhh.trim.fasta, name=*your.pmoa*.shhh.trim.names, group=*your.pmoa*.shhh.groups, dups=T)
g) Add redundant sequences back (for BLAST only):
   deunique.seqs(fasta=*your.pmoa*.shhh.trim.pick.fasta, name=*your.pmoa.*shhh.trim.pick.names)
   -- Note: the better alternative is to add weights to the representative unique sequence as discussed in my final note at the end of this file
h) Split the fasta file into separate files for each sample (barcode) using split.groups (for BLAST only):
   split.groups(fasta=*your.pmoa*.shhh.trim.pick.redundant.fasta, name=*your.pmoa*.shhh.trim.pick.names, group=*your.pmoa*.shhh.pick.groups)

4. Classify your *pmoA* using the naïve Bayesian method in mothur:

a) Use the classify.seqs command, e.g. as follows:

classify.seqs(fasta=*your.pmoa*.shhh.trim.pick.fasta, name=*your.pmoa*.shhh.trim.pick.names, group=*your.pmoa*.shhh.pick.groups, template=pmoa.mothur.2013.fasta, taxonomy=pmoa.mothur.2013.tax, method=wang, cutoff=80, processors=8)

-- Note: the fasta, name and group files are from the output of step 3f; the template and taxonomy are the files described in step 2. Do not use the redundant (3g) or split (3h) files here!

5. BLAST sequences against the *pmoA* database I have provided:

a) Set the correct paths to the BLAST program and your *pmoA* fasta files from step 3h; run megablast from the command line as follows:

   blastn -db pmoa –query *your.pmoa.sample1.*fasta -out your.pmoa.sample1.txt -num_descriptions 100 -num_alignments 100 -num_threads 8 -outfmt 0 -strand plus

   Note: the database is called pmoa, but is composed of three files (pmoa.nhr, pmoa.nin, pmoa.nsq, see step 2).

b) Perform separate blast runs for each of your preprocessed fasta files obtained using split.groups in step 3h.

6. Classify *pmoA* using LCA in MEGAN:

a) Open MEGAN. Choose Edit >> Preferences >> Use alternative taxonomy. Select the pmoa.megan.2013.tre file (step 2); make sure the pmoa.megan.2013.map file is in the same directory on your computer.
b) Choose File >> Import from BLAST and choose the megablast output and the corresponding fasta file. Configure the LCA parameters such that minimum support equals 1 and top percent equals 5. Choose apply.
c) Import the megablast output files from all your samples (Step 5b).
d) Use MEGAN to compare your samples and extract information from your data. When necessary, check the MEGAN user manual for help.

-- Note: MEGAN5 was released after submission of this manuscript and has a new feature that can factor weights for reads. Therefore, it is possible to use the dereplicated dataset obtained with mothur and to blast sequence representatives only, which will greatly speed-up the megablast query. The weights are added to the fasta header lines by writing magnitude=*number* or magnitude|*number* (see the MEGAN5 manual for details). When importing, MEGAN5 will weight each read according to the magnitude specified so that the total abundances can be compared. In mothur, run unique.seqs() and then count.seqs() to dereplicate and obtain abundances.

Please contact me if you have any problems or questions.

Marc Dumont
(m.g.dumont@gmail.com)
